# Supplementary material for: Sonographic Markers Are Useful for Detection of Early Vascular Deterioration in Children with Overweight/Obesity: Effect of a 1-Year Combined Nutritional Education and Physical Exercise Program
Source: Nutrients. 2023 Feb 10;15(4):894. doi: 10.3390/nu15040894 (PMC9959129; doi:10.3390/nu15040894)
Supplement: Supplementary file 1 [file nutrients-15-00894-s001.zip › nutrients-2158100-supplementary.pdf]

Suplementary Table S1. Baseline characteristics of the study participants attending to allocation group and sex

	CONTROL (n=29)		*p (t)*	INTERVENTION (n=148)		*p (t)*
	Boys (n=10)	Girls (n=19)		Boys (n=77)	Girls (n=71)	
Age (y)	8 ± 2	8 ± 2	0.408	8 ± 2	8 ± 2	0.820
Weight (kg)	33.3 ± 6.5	32.2 ± 6.1	0.657	41.5 ± 9.6	41.4 ± 9.5	0.930
Height (cm)	132.5 ± 9.6	129.7 ± 9.3	0.450	133.4 ± 10.2	133.9 ±11.1	0.805
BMI (z-score)	1.38 ± 0.18	1.35 ± 0.18	0.719	2.96 ± 1.05	2.61 ± 0.69	0.058
Body fat mass (kg)	7.20 ± 1.60	8.71 ± 2.49	0.096	13.14 ± 5.04	14.15 ± 4.55	0.304
Waist circumference (cm)	67.3 ± 5.0	67.5 ± 5.6	0.935	78.4 ± 9.4	77.4 ± 8.1	0.602
SBP (mmHg)	105.8 ± 8.9	107.0 ± 7.8	0.722	107.0 ± 10.7	108.5 ± 10.9	0.482
DBP (mmHG)	65.5 ± 8.0	71.7 ± 8.3	0.063	68.1 ± 8.3	69.3 ± 9.1	0.494
AST (IU/l)	30.1 ± 4.6	30.5 ± 6.6	0.875	34.0 ± 9.7	34.4 ± 9.5	0.876
ALT (IU/l)	26.7 ± 3.2	26.0 ± 8.0	0.793	31.7 ± 14.7	34.5 ± 12.9	0.310
AST/ALT ratio	1.13 ± 0.12	1.21 ± 0.26	0.340	1.16 ± 0.46	1.06 ± 0.35	0.229
GGT (IU/l)	13.2 ± 4.2	15.0 ± 6.5	0.450	16.1 ± 5.6	16.7 ± 5.1	0.714
Total cholesterol (mg/dl)	143.4 ± 21.4	155.6 ± 22.2	0.164	161.5 ± 24.5	151.4 ± 33.2	0.091
Triglycerides (mg/dl)	90.3 ± 30.9	89.5 ± 49.4	0.916	100.7 ± 47.9	117.6 ± 76.7	0.190
HDL-chol (mg/dl)	51.4 ± 6.3	56.3 ± 14.2	0.810	51,5 ± 13.5	46.9 ± 9.6	0.050
LDL-chol (mg/dl)	73.7 ± 19.6	82.1± 16.8	0.240	90.3 ± 19.7	83.4 ± 28.5	0.160
FPG (mg/dl)	85.1 ± 3.5	83.6 ± 8.4	0.604	86.8 ± 4.8	85.3 ± 5.9	0.169
Insulin (IU/ml)	5.0 ± 1.6	6.3 ± 3.0	0.217	8.0 ± 4.1	12.2 ± 12.4	**0.032**
HOMA index	1.03 ± 0.30	1.32 ± 0.67	0.201	1.72 ± 0.95	2.64 ± 3.00	0.053
Carotid IMT (mm)	0.47 ± 0.05	0.45 ± 0.05	0.540	0.50 ± 0.08	0.46 ± 0.08	**0.013**
Hepatic Shear Rate (KPa)	1.31 ± 0.14	1.38 ± 0.17	0.289	1.27 ± 0.17	1.38 ± 0.23	**0.012**

Data represent mean ± sd. Differences were analysed by Student's t test.

Supplementary Table S2. Anthropometrical and clinical characteristics of the participants at baseline and at the end of the intervention

	CONTROL (n=29)				INTERVENTION (n=148)				
	Baseline	End of intervention	p	Treatment effect	Baseline	End of intervention	p	Treatment Effect	Effect Size (partial η2)
Weight (kg)	32.6 ± 6.1	35.1 ± 6.6	<0.001	2.5	41.5 ± 9.5	43.8 ± 10.3	<0.001	2.3	0.636
BMI (z-score)	1.36 ± 0.18	1.27 ± 0.31	0.033	-0.09	2.80 ± 0.91	2.44 ± 0.80	<0.001	-0.36	0.004
Body fat mass (kg)	8.19 ± 2.31	8.13 ± 2.33	0.846	-0.06	13.61 ± 4.82	13.47 ± 4.76	0.214	-0.14	0.005
Waist circumference (cm)	67.4 ± 5.3	70.0 ± 5.4	<0.001	2.6	77.9 ± 8.7	80.3 ± 9.2	<0.001	2.4	0.340
SBP (mmHg)	106.6 ± 8.1	104.1 ± 11.0	0.354	-2.5	107.7 ± 10.8	109.5 ± 10.4	0.408	1.8	0.001
DBP (mmHG)	69.6 ± 8.6	63.8 ± 8.4	0.003	-5.8	68.7 ± 8.7	68.2 ± 6.5	0.002	-0.5	0.083
AST (IU/l)	30.3 ± 5.9	32.2 ± 5.6	0.180	1.9	34.1 ± 9.6	33.0 ± 8.7	0.374	-1.1	0.001
ALT (IU/l)	26.2 ± 6.7	24.0 ± 6.0	0.037	-2.2	33.0 ± 13.9	30.5 ± 13.4	0.267	-2.5	0.014
AST/ALT ratio	1.18 ± 0.22	1.38 ± 0.28	0.002	0.20	1.12 ± 0.41	1.18 ± 0.40	0.399	0.06	0.047
GGT (IU/l)	14.3 ± 5.8	15.6 ± 3.2	0.165	1.3	16.9 ± 5.4	18.4 ± 4.9	0.012	1.5	0.046
Total cholesterol (mg/dl)	151.4 ± 22.3	156.7 ± 29.3	0.152	5.3	156.8 ± 29.1	158.8 ± 26.9	0.664	2.0	0.012
Triglycerides (mg/dl)	90.1 ± 43.3	79.9 ± 32.9	0.163	-10.2	108.5 ± 63.1	91.1 ± 42.6	0.012	-17.4	0.040
HDL-chol (mg/dl)	54.6 ± 12.2	58.5 ± 11.9	0.047	3.9	49.3 ± 12.0	51.5 ± 12.2	0.129	2.2	0.044
LDL-chol (mg/dl)	79.2 ± 17.9	82.3 ± 23.5	0.376	3.1	87.1 ± 24.3	89.4 ± 22.3	0.458	2.3	0.010
FPG (mg/dl)	84.1 ± 7.1	85.6 ± 6.1	0.322	1.5	86.1 ± 5.3	86.2 ± 6.1	0.843	0.1	0.006
Insulin (IU/ml)	5.8 ± 2.7	7.6 ± 2.4	0.002	1.8	9.9 ± 9.1	11.4 ± 7.1	0.247	1.5	0.022
HOMA index	1.22 ± 0.58	1.62 ± 0.56	0.002	0.40	2.15 ± 2.19	2.45 ± 1.62	0.295	0.30	0.019
Carotid IMT (mm)	0.46 ± 0.05	0.36 ± 0.06	<0.001	-0.10	0.48 ± 0.08	0.37 ± 0.06	<0.001	-0.11	0.503
Hepatic Shear Rate (KPa)	1.35 ± 0.16	1.15 ± 0.12	<0.001	-0.2	1.32 ± 0.21	1.20 ± 0.17	<0.001	-0.12	0.306

Data represent mean ± sd. Differences between end of treatment and baseline values were evaluated by mean of an ANOVA analysis.
